# π Berry phase and Zeeman splitting of Weyl semimetal TaP

**DOI:** 10.1038/srep18674

**Published:** 2016-01-04

**Authors:** J. Hu, J. Y. Liu, D. Graf, S. M. A. Radmanesh, D. J. Adams, A. Chuang, Y. Wang, I. Chiorescu, J. Wei, L. Spinu, Z. Q. Mao

**Affiliations:** 1 Department of physics and Engineering Physics, Tulane University, New Orleans, Louisiana 70118, USA; 2 National High Magnetic Field Laboratory, Florida State University, Tallahassee, Florida 32310, USA; 3 Advanced Materials Research Institute and Department of Physics, University of New Orleans, New Orleans, Louisiana 70148, USA; 4 Department of Physics, Florida State University, Tallahassee, Florida 32306, USA

## Abstract

The recent breakthrough in the discovery of Weyl fermions in monopnictide semimetals provides opportunities to explore the exotic properties of relativistic fermions in condensed matter. The chiral anomaly-induced negative magnetoresistance and π Berry phase are two fundamental transport properties associated with the topological characteristics of Weyl semimetals. Since monopnictide semimetals are multiple-band systems, resolving clear Berry phase for each Fermi pocket remains a challenge. Here we report the determination of Berry phases of multiple Fermi pockets of Weyl semimetal TaP through high field quantum transport measurements. We show our TaP single crystal has the signatures of a Weyl state, including light effective quasiparticle masses, ultrahigh carrier mobility, as well as negative longitudinal magnetoresistance. Furthermore, we have generalized the Lifshitz-Kosevich formula for multiple-band Shubnikov-de Haas (SdH) oscillations and extracted the Berry phases of π for multiple Fermi pockets in TaP through the direct fits of the modified LK formula to the SdH oscillations. In high fields, we also probed signatures of Zeeman splitting, from which the Landé *g*-factor is extracted.

In Dirac semimetals, the discrete band touching points near the Fermi level - the Dirac nodes - are protected from gap opening by crystalline symmetry. The linear energy band dispersion near Dirac nodes hosts Dirac fermions whose low energy physics can be described by Dirac equation. With lifting spin degeneracy by breaking either time-reversal or inversion symmetry, a Dirac node is expected to split to a pair of Weyl nodes with opposite chirality^1^,^2^. The conservation of chirality of such pairs of separated Weyl nodes in momentum space leads to their topological robustness against translational symmetry invariant perturbations^1^,^2^. Near Weyl nodes, electrons behave as Weyl fermions and result in exotic signatures in Weyl semimetal, such as surface Fermi arcs that connect Weyl nodes of opposite chirality^3^. Moreover, Weyl fermions in Weyl semimetals also manifest themselves with exotic signatures in electron transport, such as chiral anomaly-induced negative longitudinal magnetoresistance (Adler-Bell-Jackiw chiral anomaly) and Berry phase of π.

The recently proposed Weyl semimetal phase in transition metal monopnictides TX (T = Ta/Nb, X = As, P)^1^,^2^ has stimulated intensive interests. Unlike the proposed magnetic Weyl semimetals^3^,^4^,^5^, Weyl nodes appearing in TX is due to broken inversion symmetry^1^,^2^. Discontinuous surface Fermi arcs has been observed in photoemission spectroscopy studies on these materials^6^,^7^,^8^,^9^,^10^, confirming the existence of Weyl nodes. In addition, the Adler-Bell-Jackiw chiral anomaly, which is reflected as negative longitudinal magnetoresistance (MR)^11^,^12^,^13^, has also been observed in TaAs^14^,^15^,^16^, TaP^17^,^18^, NbAs^19^, and NbP^20^.

In additional to the chiral anomaly-induced negative MR, non-trivial Berry phase of π is another important characteristic which can be revealed in the transport measurements. Berry phase describes the additional geometrical phase factor acquired in the adiabatic evolution along a closed trajectory in the parameter space^21^. Such additional phase does not depend on the details of the temporal evolution and thus differs from the dynamical phase^21^,^22^. In condensed matter, the Berry phase is determined by the topological characteristics of the electron bands in the Brillouin zone^22^,^23^,^24^. A non-zero Berry phase reflects the existence of band touching point such as Dirac nodes, and manifest itself in observable effects in quantum oscillations. The cyclotron motion (that is, closed trajectory in momentum space) of Dirac fermions under magnetic field *B* induces Berry phase that changes the phase of quantum oscillations. Generally, through mapping the Landau level (LL) fan diagram (*n*^th^ LL index *vs.* 1/*B*_*n*_, the inverse of the applied magnetic field), the Berry phase can be conveniently extracted from the intercept of the linear extrapolation of LL index to the zero of inverse field 1/*B*. Experimentally, a Berry phase of π arising from the linear band dispersion of a Dirac cone^22^,^24^ has been probed from the Shubnikov–de Haas (SdH) oscillations in both two dimensional (*e.g.* graphene^25^,^26^, topological insulators^27^,^28^, and SrMnBi_2_^29^) and three dimensional (*e.g.* Cd_3_As_2_^30^,^31^) Dirac fermion systems.

In Weyl semimetals, a similar linear band dispersion^1^,^2^,^6^,^7^,^8^,^9^,^10^ also generates a non-trivial Berry phase of π. However, unlike the well-established π Berry phase in Dirac systems as mentioned above^25^,^26^,^27^,^28^,^29^,^30^,^31^, the experimental determination of Berry phase using the LL fan diagram remains elusive for monopnictide Weyl semimetals. First, the identification of the integer LL indices is inconsistent among published works; both the resistivity minima^19^ and maxima^14^,^15^,^16^,^20^ have been used to assign the integer LL indices. Secondly, while all of these reports claim a non-trivial or π Berry phase, the intercept values obtained from the LL index plot, which are used to derive Berry phase, are diverse: 0^14^,^15^ and −0.08^16^ for TaAs, 0.12^19^,^32^ for NbAs, and 0.32^20^ for NbP. As for TaP, however, Berry phase remains unexplored in the earlier studies^17^,^18^. Indeed, the LL fan diagram technique may not be an efficient method to extract the precise value of Berry phase in these monopnictide Weyl semimetals. Unlike previously studied Dirac systems which display only single frequency in SdH oscillations^25^,^26^,^27^,^28^,^29^,^30^,^31^, monopnictide Weyl semimetals exhibit quantum oscillations with multiple frequencies due to the existence of multiple Fermi pockets^1^,^2^,^6^,^7^,^8^,^9^,^10^. Therefore the oscillation peaks may not accurately correspond to the LL indices due to wave superposition. In addition, if one oscillation frequency is close to another (which is commonly seen in these systems for some certain field orientations^17^,^20^,^32^), separating the individual peak requires high magnetic field which was not achieved in previous low field studies^14^,^15^,^16^,^19^,^20^.

In order to address these issues, we conducted systematic high field magnetotransport measurements on TaP single crystals up to 31 T. Our TaP crystal used in this study has superior quality, as demonstrated in the extremely large MR (1 × 10^6^% at 1.6 K and 31 T, 300% at 300 K and 9 T) and ultra-high mobility (2.5 × 10^6^ cm^2^/V s for hole-type carriers at 1.6 K). The availability of such high quality crystals allows us to probe intrinsic quantum transport properties of TaP. Instead of using the LL fan diagram technique to extract the Berry phase, we have generalized the Lifshitz-Kosevich (LK) formula^33^,^34^,^35^ and fitted the multi-frequency SdH oscillations in TaP. This approach is capable of revealing Berry phases for multiple Fermi pockets. Through this approach, we find that electrons from multiple Fermi pockets have π Berry phases accumulated along their cyclotron orbits, which agrees well with the nature of Weyl fermions. In high fields, we also observed Zeeman spin splitting, which enables us to extract the Landé *g*-factor for TaP for the first time.

## Results and Discussions

**Figure 1:**
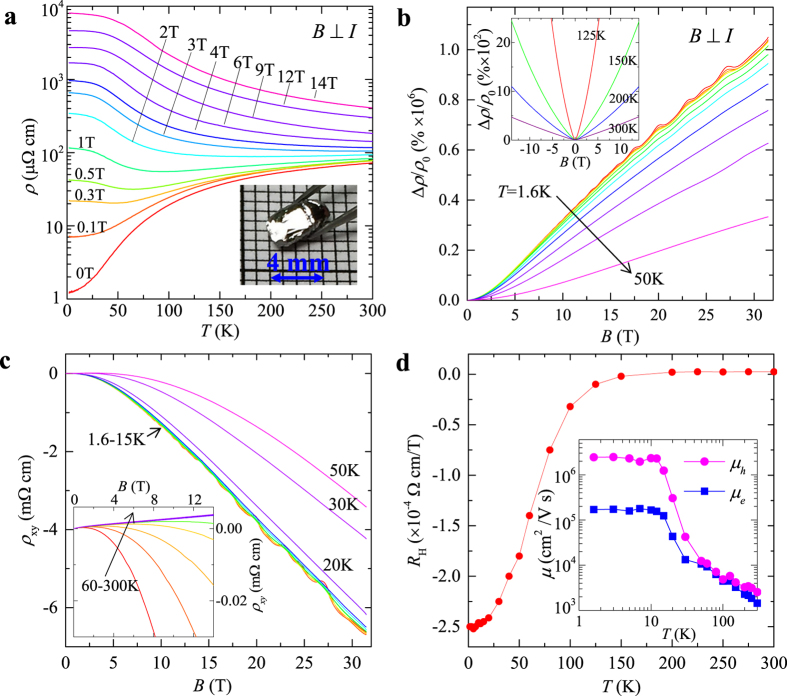
Transport properties of TaP. (**a**) Temperature dependence of resistivity under various magnetic field *B* from 0 to 14 T. The magnetic field is applied perpendicual to the current direction. Inset: image of a large TaP crystal. (**b**) Magnetoresistance Δ*ρ* /*ρ*_0_ = [*ρ*(*B*)–*ρ*(*B* = 0)]/*ρ*(*B* = 0) of TaP at various temperatures *T*. From bottom to top: *T* = 50, 40, 30, 20, 15, 12, 10, 7, 5, 3, and 1.6 K. Inset: Magnetoresistance at high temperatures. (**c**) Hall resistivity *ρ*_*xy*_ at various temperatures. The inset shows the *ρ*_*xy*_ at higer temperature from 60 K to 300 K (**d**) Temperature dependence the Hall coefficient *R*_H_.The inset shows the mobility extracted from the two-band model fitting (see text).

Figure 1 shows the magnetotransport properties of TaP with magnetic field along the crystallographic [100] axis and perpendicular to the current direction. The zero-field resistivity of TaP displays metallic behavior, with residual resistivity ~1.2 μΩ cm at 2 K and the residual resistivity ratio (RRR) *ρ*(300 K)/*ρ*(2 K) ~65, implying high quality of our single crystals. Applying magnetic field perpendicular to the current direction (*B*⊥*I*) induces extremely large MR and changes the temperature dependence of resistivity dramatically. A resistivity upturn appears when field *B* > 0.1 T, which becomes more significant with increasing magnetic field and develops to an insulting-like behavior in full temperature range when *B* > 3 T (Fig. 1a). Such “metal - to - insulator-like” evolution driven by magnetic field is also observed in WTe_2_^36^ and consistent with pervious observations in TaP^18^ and other Weyl semimetals^14^,^15^,^16^,^19^,^37^,^38^.

The extremely large MR can be better visualized in the field dependence of resistivity presented in Fig. 1b. At *T* = 1.6 K, the normalized MR Δ*ρ*_*xx*_/*ρ*_*0*_ ( = [*ρ*_*xx*_(B) − *ρ*_*xx*_(B = 0)]/*ρ*_*xx*_(B = 0)) reaches 3 × 10^5^% at 9 T, larger than pervious observations in ref. 17. Further increasing field leads MR to reach 1 × 10^6^% at 31 T without saturation, and SdH oscillations become visible above 9 T. Even at room temperature, giant MR as large as 300% can be observed at *B* = 9 T (Fig. 1b, inset). It is surprising to observe such huge MR in a sample with RRR ~ 65, given that large MR is usually accompanied with RRR of a few hundreds or even thousands^36^,^39^,^40^. In fact, large MR with RRR less than 100 is commonly observed in monopnictide Weyl semimetals^14^,^15^,^16^,^17^,^18^,^19^,^20^,^32^,^37^,^38^, in contrast with Dirac semimetal Cd_3_As_2_ in which MR and RRR are more closely related^41^. In addition to the large magnitude, a linear field dependence for MR (MR∝*B*) is also widely reported in monopnictide Weyl semimetals^14^,^15^,^16^,^18^,^19^,^20^,^37^. In our TaP single crystals, following a conventional quadratic dependence in low fields, a similar linear MR is also observed from *B* = 3 T up to 16 T. Nevertheless, in the high field region which was not well explored in the previous studies, a power law dependence ~*B*^0.65^ is found to better describe the MR data, suggesting that additional transport mechanisms may occur in high field.

To further characterize the electronic properties of TaP, we have measured the Hall resistivity *ρ*_*xy*_ up to high fields. As shown in Fig. 1c, at low temperatures, the negative slop of linear field dependence of *ρ*_*xy*_(*B*) in high field range indicates that the transport is dominated by electrons; whereas the appearance of the nonlinear curvature in low field region implies the involvement of hole-type carriers. Indeed, both the theoretical calculations^1^,^2^ and photoemission studies^6^,^7^,^8^,^9^,^10^ reveal both electron- and hole- Fermi surfaces in monopnictide Weyl semimetals. With increasing temperature, the hole-type carriers gradually dominate the transport above 150 K, as indicated by the positive slop of *ρ*_xy_(B) at high field (Fig. 1c, inset). Such evolution leads to the sign change of Hall coefficient *R*_H_ that was derived from the linear dependence of *ρ*_xy_(*B*) at high field (Fig. 1d), consistent with the previous reports on monopnictide Weyl semimetals^14^,^15^,^16^,^17^,^18^,^19^,^20^,^37^,^38^.

From two-band model fitting^42^, we have obtained ultra-high mobility *μ*_e_ ~ 1.8 × 10^5^ cm^2^/V s and *μ*_h_ ~ 2.5 × 10^6^ cm^2^/V s at 1.6 K for electrons and holes respectively, as well as a nearly ideal electron and hole compensation with *n*_*e*_ ~ 11.53 × 10^18^ cm^−3^ and *n*_*h*_ ~ 11.50 × 10^18^ cm^−3^. The observed high mobility is larger than that in previous observations in TaP (e.g., *μ*_h_ ~ 9 × 10^4^ cm^2^/Vs^17^ and 2 × 10^5^ cm^2^/Vs^18^), and comparable to that of NbP–the largest mobility seen in the monopnictide Weyl semimetal family (e.g., 5 × 10^6^ 38 and 1 × 10^7^ cm^2^/V s^20^, estimated by single band model). Such high mobility may be responsible for the rapid increase of resistivity with magnetic field as described by *ρ* ~ (*μB*)^2^, while the nearly ideal electron-hole compensation prevents the saturation of MR in high fields^36^. However, this simple classical model fails to interpret the linear and ~*B*^0.65^ dependence of MR at high fields, suggesting the involvement of other mechanisms such as quantum linear MR^43^ or plausible broken topological protection under magnetic field^41^.

**Figure 2:**
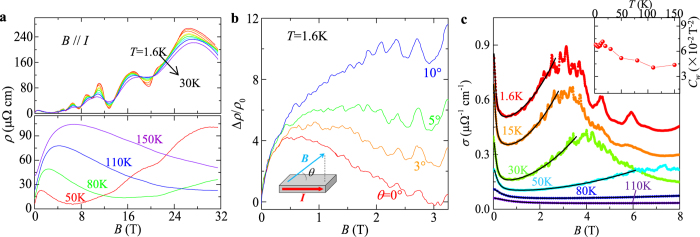
Chiral anomaly-induced negative MR in TaP. (**a**) the longitudinal MR at 1.6–30 K (top panel) and 50–150 K(bottom panel). (**b**) longitudinal MR at 1.6 K at different field orientation. The inset shows the experiment setup. (**c**) Fitting of the magnetoconductivity *σ*(*B*) at different temperatures using Eq. 1 (see text). Data for different temperatures has been shifted for charlity. The inset shows the temperature dependence of *C*_w_ extracted form fitting (see text).

In Weyl semimetals, the pair of Weyl nodes acts as source and drain of Berry flux, leading to a non-zero Berry curvature **Ω**_p_ and causing an additional topological contribution to the Weyl fermions dynamics, which is proportional to the product of electric and magnetic field, *i.e.*, ∝(***E**·**B***)**Ω**_p_^12^,^13^. As a consequence, non-orthogonal electric and magnetic field (***E**·**B*** ≠ 0) can lead to charge transfer between two Weyl nodes with opposite chirality. Such violation of the chiral charge conservation is known as the Adler-Bell-Jackiw anomaly or chiral anomaly^11^,^12^,^13^, and results in negative MR that can take place in semiclassical regime^12^,^13^. As shown in Fig. 2a,b, with magnetic field aligned parallel to the current direction (*B*//*I*), we observed negative longitudinal MR above *B* = 0.5 T at 1.6 K, which can be quickly suppressed when the field orientation is deviated from the current (Fig. 2b), in agreement with the pervious observations in Weyl semimetals^14^,^15^,^16^,^17^,^18^,^19^. The positive MR below 0.5 T may be ascribed to weak antilocalization due to strong spin-orbit coupling. With increasing field above 4 T, the negative MR turns to be positive, which roughly increases with *B*^2^ and is accompanied by strong SdH oscillations. This suggests a dominating classical orbital MR at high fields which is probably induced by non-ideal parallel alignment between *B* and *I*, or Fermi surface anisotropy^44^. With the suppression of the classic MR by rising temperature, negative MR becomes more prominent and extends to high field up to 31 T, without any signature of saturation (see the bottom panel in Fig. 2a). Such results are consistent with the earlier low field experiments for TaP^17^, and provide the first direct observation of significant chiral anomaly contribution to transport at high fields. The strong negative MR at high temperatures suggests that Weyl fermions make more significant contributions in TaP as compared to other monopnictide Weyl semimetals. This is consistent with the recent photoemission study which found that four pairs of Weyl points are located at the Fermi energy^10^.

Generally, in the quantum limit where all electrons are condensed to the lowest Landau level (LL), only the LL = 0 chiral branch is occupied and the intra-node scattering is prohibited. Therefore, the longitudinal current (***E***//***B***) can be relaxed only via inter-node scattering between a pair of Weyl nodes, causing the enhanced negative MR^11^,^12^. Indeed, a previous study has found that the negative MR in TaP is enhanced upon cooling and persists up to 14 T^17^, in sharp contrast with our observations of the high field (>4 T) positive MR at 1.6 K (Fig. 2a). This can be understood in terms of the ultra-high mobility of our TaP single crystals (an order of magnitude larger than that in ref. 17), which gives rise to much stronger classical MR since MR∝*μ*^2^*B*^2^.

More quantitative information of Weyl fermions transport in TaP can be extracted through their *B*^2^-dependence of magnetoconductivity (MC)^12^,^13^. The total conductivity can be written as 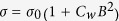 in parallel field, in which *σ*_0_ is the normal conductivity and the chiral anomaly contribution is thus to be *σ*_0_*C*_*w*_*B*^2^ 11. In our sample, the presence of weak antilocalization leads to correction to normal conductivity, *i.e.*, 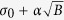
45. With the consideration of the additional classic positive MR ∝*B*^2^ 45, the MC of TaP can be written as:


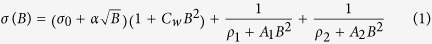


Similar to the previous reports, two classic MR terms are necessary to obtain the satisfactory fit^15^,^17^. As shown in Fig. 2c, the above equation fits the chiral anomaly induced positive MC (negative MR) very well for all measured temperatures, as shown by the black solid fitting curves in Fig. 2c. However, the high field negative MC (positive MR) at low temperatures cannot be simultaneously described by Eq. 1. Similar observation has also been reported in ref. 17. This can be understood in terms of approaching quantum limit at low temperatures, where the contribution from chiral anomaly becomes linearly dependent on *B*^11^,^12^,^13^.

From the fitting we have extracted the chiral anomaly contribution *σ*_w_ = *σ*_0_*C*_w_*B*^2^ 11 at various temperatures. In principle, *C*_w_ is *T*-independent and the temperature variation of *σ*_w_ results from *σ*_0_(*T*) (∝*μ*(*T*)). As expected, *C*_w_ displays very weak temperature dependence, as shown in the inset of Fig. 2c.

In addition to the chiral anomaly induced negative MR, the Berry phase of π accumulated in the cyclotron motion of Weyl fermions is another fundamental topological property of Weyl semimetals, since that the Berry phase takes the value of 0 and π for parabolic and linear band dispersions, respectively^24^. The effect of this additional phase factor in SdH oscillation can be described by the Lifshitz-Kosevich (LK) formula which is developed for 3D system with arbitrary band dispersions^33^,^34^,^35^:


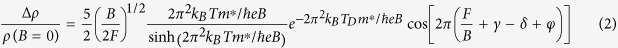


In Eq. 2, the hyperbolic and exponential terms describe the temperature and field damping of oscillation amplitude, which are determined by effective mass m* and Dingle temperature *T*_*D*_ respectively. The oscillation frequency is described by the cosine term that contains a phase factor 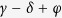, in which 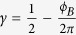 and *ϕ*_B_ is Berry phase. The phase shift *δ* is determined by the dimensionality of Fermi surface and takes values 0 for 2D and ±1/8 for 3D cases. The additional phase factor *φ*, which is missing in the original LK formula^33^,^34^,^35^, is due to the relative phase between conductivity and resistivity oscillations, i.e. Δ*σ* and Δ*ρ*^46^. Given that 
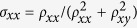
, when the longitudinal resistivity *ρ*_*xx*_ ≫ transverse (Hall) resistivity *ρ*_*xy*_, the oscillation component of *σ*_*xx*_ obtained by taking the derivative is 
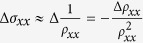
, indicating completely out-of- phase for Δ*σ* and Δ*ρ*. In contrary, when *ρ*_*xx*_ ≪ *ρ*_*xy*_, Δ*σ* and Δ*ρ* are in phase since 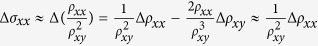. Therefore, when *σ*_*xx*_ is maximized with lifting LL to Fermi energy, *ρ*_*xx*_ can display either minimum (*ρ*_*xx*_  ≫ *ρ*_*xy*_) or maximum (*ρ*_*xx*_ ≪ *ρ*_*xy*_), leading to an additional phase factor *φ* = 1/2 or 0, respectively, in the LK formula.

**Figure 3:**
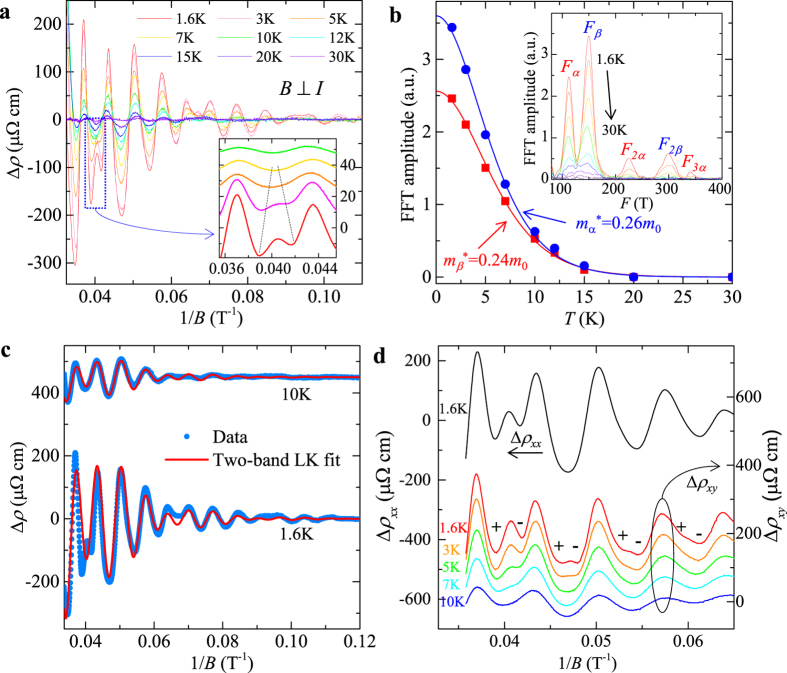
SdH oscillations when *B*⊥*I*. (**a**) The oscillation component of resistivity at different temperatures, obtained by Δ*ρ* = *ρ* − *ρ*_*br*_. The inset shows the Zeeman splitting of an oscillation peak at high field, which gradually merges with raising temperature. Data at different temperatures has been shifted for clarity. (**b**) The temperature dependence of FFT amplitude for Δ*ρ*/*ρ* (*B* = 0). The solid lines show the effective mass fitting according to the temperature damping term of the Lifshitz-Kosevich formula. The inset shows the FFT for Δ*ρ*/*ρ*(*B* = 0) at various temperatures. (**c**) Fitting of SdH oscillation at 1.6 K and 10 K to the generalized LK model. The 10 K data has been shifted for clarity. (**d**) Zeeman splitting of longitudinal (Δ*ρ*_*xx*_) and Hall resistivity (Δ*ρ*_*xy*_). Δ*ρ*_*xy*_ at different temperatures has been shifted for clarity.

**Figure 4:**
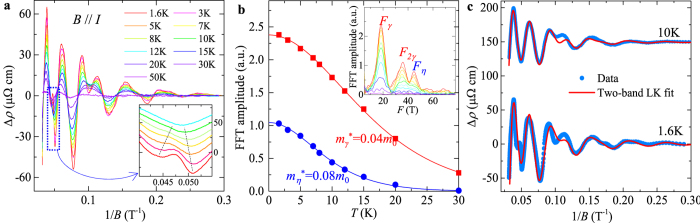
SdH oscillations when *B*//*I*. (**a**) The oscillation component of resistivity Δ*ρ* = *ρ*–*ρ*_*br*_ at different temperatures. The inset shows the Zeeman splitting of an oscillation peak at high field, which gradually merges with raising temperature. Data at different temperatures has been shifted for clarity. (**b**) The temperature dependence of FFT amplitude for Δ*ρ*/*ρ* (*B* = 0). The solid lines show the effective mass fitting according to the temperature damping term of the Lifshitz-Kosevich formula. The inset shows the FFT for Δ*ρ*/*ρ* (*B* = 0) at various temperatures (1.6–50 K). (**c**) Fitting of SdH oscillations at 1.6 K and 10 K to the generalized LK model. The 10 K data has been shifted for clarity.

Based on the above analysis, resistivity exhibits maximum (*ρ*_*xx*_ ≪ *ρ*_*xy*_) or minimum (*ρ*_*xx*_ ≫ *ρ*_*xy*_) with the Fermi level successively reaching the *n*^th^ LL. Therefore from Eq. 2 we can obtain the Lifshitz-Onsager quantization rule: 
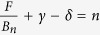
 (when *ρ*_*xx*_ ≪ *ρ*_*xy*_) or 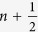 (when *ρ*_*xx*_ ≫ *ρ*_*xy*_). Such linear relation between 1/*B*_*n*_ and *n* allows the extraction of 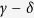 and consequently the Berry phase through the intercept of the LL fan diagram (in fact, the intercept is the phase factor *γ* *–* *δ* *+* *φ* in the LK formula), as seen in a variety of Dirac systems^25^,^26^,^27^,^28^,^29^,^30^,^31^. However, the LL fan diagram approach is less efficient in monopnictide Weyl semimetals due to multiple frequencies involved in oscillations as discussed above. In Fig. 3a we show the oscillatory component of resistivity Δ*ρ* for *B*⊥*I* at various temperatures obtained by subtracting the smooth MR background (Δ*ρ* = *ρ* − *ρ*_*br*_). A clear splitting can be observed around 0.04 T^−1^ (*B* = 25 T), which disappears above 10 K (Fig. 3a, inset) and can be ascribed to Zeeman splitting and will be discussed later. Except for this feature, Δ*ρ* is, however, still not exactly periodic in 1/*B*. In fact, the fast Fourier transfer (FFT) of Δ*ρ* (Fig. 3b, inset) yields two major frequencies at *F*_*α*_ = 110 T and *F*_*β*_ = 150 T with comparable amplitude (the high field splitting data is not included for FFT). The superposition of two oscillation waves could cause broadening and even shift of the oscillation extrema, making it is difficult to assign LL index. Similar situation also occurs for the case of *B*//*I* (Fig. 4a,b), in which major frequencies of *F*_*γ*_ = 18 T and *F*_*η*_ = 44 T can be resolved via FFT.

Similar multi-frequency problem also occurs in other monopnictide Weyl semimetals due to their multiple Fermi pockets. Most of earlier works focused on the “major” oscillation extrema and found the corresponding Berry phase for one Fermi pocket^14^,^16^,^19^,^20^. Another approach is to use the 2^nd^ derivative of Δ*ρ* to separate oscillation peaks for different frequencies^32^. In those works^14^,^16^,^19^,^20^,^32^, a variety of quite different intercepts in LL fan diagram has been obtained as mentioned above. Such inconsistence may be associated with sample variation or, more likely, the uncertainty in identifying 1/*B*_n_ due to the superposition of oscillation peaks.

In TaP, Berry phase has not been explored in previous magnetotransport studies^17^,^18^. In order to extract the accurate Berry phase in TaP, instead of the LL fan diagram, we attempted to use the LK formula (Eq. 2) to fit the multi-frequency SdH oscillations directly. Given that we have observed two major frequencies for both *B*⊥*I* and *B*//*I*, we can reasonably assume that the total resistivity *ρ* due to two Fermi pockets follows 1/*ρ* = 1/*ρ*_1_ + 1/*ρ*_2_. Taking the differential we can obtain 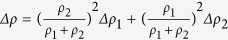, indicating that the resistivity oscillations are additive and the LK formula (Eq. 2) can be easily generalized for multi-frequency oscillations by linear superposition. To reduce the fitting parameters, the effective mass m^*^ can be first extracted through the fit of the temperature dependence of the amplitude of FFT for Δ*ρ*/*ρ*_0_ to the thermal damping term in the LK formula, *i.e.*, 
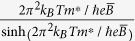
 with 1/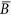 being the average inverse field. The values of m^*^ are considerably smaller for *γ* and *η* Fermi pockets (*m*_*γ*_ = 0.04 *m*_0_ and *m*_*η*_ = 0.08 *m*_0_, *m*_0_ is the free electron mass, Fig. 4b) than those of *α* and *β* Fermi pockets (*m*_*α*_ = 0.26 *m*_0_ and *m*_*β*_ = 0.24 *m*_0_, Fig. 3b), consistent with the previous results^17^.

To avoid the influence from Zeeman splitting, during the fitting using the LK formula, we have fitted the SdH oscillations at 10 K where the Zeeman effect is minimized by the thermal broadening of LL (see insets of Figs 3a and 4a), as well as the low temperature (*T* = 1.6 K) data with the high field splitting being excluded. As shown in Figs 3c and 4c, the two-band LK formula reproduces the resistivity oscillations well. The phase shift *γ – δ + φ* = 
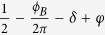
 extracted from fitting are 0.42(3) and 0.41(2) for *α* and *β* Fermi pockets probed when *B*⊥*I*, and 0.12(3) and 0.67(6) for *η* and *γ* Fermi pockets probed when *B*//*I*. Given that *ρ*_*xx*_ ≫ *ρ*_*xy*_ for both *B*⊥*I* and *B*//*I* (*ρ*_xy_ = 0 for *B*//*I*) in our experiments, we take *φ* = 1/2 as discussed above. With this condition, the phase shift obtained from our two-band LK fits yields a Berry phase *ϕ*_B_ close to π for *α*, *β*, and *γ* Fermi pockets (in particular, 0.91(±0.06)π, 0.93(±0.04)π, and 0.91(±0.12)π for *α*, *β*, and *γ* Fermi pockets respectively), as well as a trivial Berry phase near zero for the *η* Fermi pocket (0.1π). Any other trails with the fixed zero Berry phase cannot yield a better fit, confirming the existence of the non-zero Berry phase. Such a result is consistent with the linear band dispersion of Weyl fermions in TaP and previous LL fan diagram analyses for other monopnictide Weyl semimetals^14^,^15^,^16^,^19^,^20^,^32^, which revealed a non-trivial Berry phase for single Fermi pocket. Given the effectiveness of our two-band LK fit, the generalization of our approach would allow determination of Berry phases of any multiple band systems with relativistic fermions for which the LL fan diagram approach is less efficient as discussed above.

In addition to the Berry phase, with high field measurements we determined the Landé *g*-factor through Zeeman splitting for the first time. As stated above, we have observed peak splitting in high field oscillations (around 0.04 T^−1^ for *B*⊥*I* and 0.047 T^−1^ for *B*//*I*, see Figs 3a and 4a). With raising temperature above 10 K, the splitting gradually merges to single peak (insets of Figs 3a and 4a), implying an origin of the Zeeman effect. Such splitting becomes more significant in the oscillation of Hall resistivity Δ*ρ*_*xy*_, and also weakens with raising temperature, as shown in Fig. 3d. Unfortunately, we are unable to separate electrons from different Fermi surfaces in SdH oscillations, but an average *g*-factor of 2–2.9 can be obtained for *α* and/or *β* Fermi pockets, and 5.5–6.7 for *η* and/or *γ* Fermi pockets, following the method used in ref. 31.

## Conclusion

In summary, we have preformed systematic high field magnetotransport measurements on high quality TaP single crystals, which display extremely large magnetoresistance and ultra-high mobility. We have found the chiral anomaly-induced negative MR can extend up to 31T at high temperatures, implying significant involvement of Weyl fermions in transport. More importantly, using the generalized two band LK formula, we have successfully determined the non-trival Berry phase very close to π for multiple Fermi pockets in TaP. Such results not only reveal the unexplored fundamental topological properties of TaP, but also provide an effective approach that can be generalized to other Weyl semimetals.

Note: In the preparation of the manuscript, we were aware of a high field measurement which revealed quantum phase transition in TaP^47^, and a low field magnetotrasport work which revealed a non-trivial Berry phase for a single Fermi pocket in TaP using LL fan diagram^48^.

## Methods

### Single crystal growth and characterization

The high quality TaP single crystals were synthesized using a chemical vapor transport technique. The stoichiometric mixture of Ta and P powder was sealed in a quartz tube with iodine as transport agent (20 mg/cm^3^). Convex polytopes-like single crystals with metallic luster as large as 4 mm × 2.5 mm × 2 mm (Fig. 1a, inset) can be obtained via the vapor transport growth with a temperature gradient from 950 °C to 850 °C. The composition and structure of TaP single crystals were checked by X-ray diffraction and Energy-dispersive X-ray spectrometer.

### Magnetotransport measurements

Samples for transport measurements were polished to be plate-like with [100] surface. The magnetotransport measurements are performed using a 14 T Physics Property Measurement System at AMRI, UNO, and the 31 T resistive magnets at National High Magnetic Field Laboratory (NHMFL) in Tallahassee.

## Additional Information

**How to cite this article**: Hu, J. *et al.* π Berry phase and Zeeman splitting of Weyl semimetal TaP. *Sci. Rep.* **6**, 18674; doi: 10.1038/srep18674 (2016).
